# Maximizing expectancy violation and exposure outcomes in patients with PTSD

**DOI:** 10.1080/20008066.2024.2447183

**Published:** 2025-01-07

**Authors:** Marike J. Kooistra, Maartje Schoorl, Danielle A. C. Oprel, Willem van der Does, Rianne A. de Kleine

**Affiliations:** a Department of Clinical Psychology, Leiden University, Leiden, The Netherlands; b Parnassia Groep, PsyQ, The Hague, The Netherlands; c Leiden University Treatment Center (LUBEC), Leiden, The Netherlands

**Keywords:** Posttraumatic stress disorder, exposure therapy, expectancy violation, inhibitory learning, mechanisms of change, Trastorno de estrés postraumático, terapia de exposición, violación de expectativas, aprendizaje inhibitorio, mecanismos de cambio

## Abstract

**Background:** It has been proposed that maximizing expectancy violation enhances the efficacy of exposure therapy. The clinical utility of expectancy violation remains unclear and it has not yet been studied in PTSD.

**Objective:** We aimed to test whether explicitly focusing on expectancy violation leads to superior exposure outcomes.

**Method:** Adult treatment-seeking patients with PTSD (*N* = 60) were randomly assigned to one 90-minute exposure session focusing on either expectancy violation or a control condition without an expectancy focus. Assessments occurred before the session and one week later, measuring changes in fear responses during a script-driven imagery task, and PTSD symptoms.

**Results:** Using multilevel analyses, we found no between-condition differences. On average, fear responses to the imagery and PTSD symptoms decreased over time. The expectancy violation condition exhibited a greater decrease in threat appraisal, which appeared to mediate symptom reduction.

**Conclusions:** We found no evidence that explicitly focusing on expectancy violation led to superior immediate effects. However, it may lead to more changes in expectancies which could affect symptom improvement over an extended period. Further research is needed to determine whether emphasizing expectancy violation in exposure therapy for PTSD is advantageous.

## HIGHLIGHTS

- Drawing attention to expectancy violation did not lead to more PTSD symptom reduction.
- Threat appraisals decrease more when expectancies are explicitly violated.
- A shift in expectancies may contribute to PTSD symptom reduction.
- It is uncertain whether applying the inhibitory retrieval model benefits exposure therapy for PTSD.

## Abstract (es)

**Antecedentes:** Se ha propuesto que maximizar la violación de expectativas mejora la eficacia de la terapia de exposición. La utilidad clínica de la violación de expectativas sigue sin estar clara y aún no se ha estudiado en el TEPT.

**Objetivo:** Nuestro objetivo era comprobar si centrarse explícitamente en la violación de expectativas conduce a mejores resultados de exposición.

**Método:** Pacientes adultos con TEPT que buscaban tratamiento (*N* = 60) fueron asignados aleatoriamente a una sesión de exposición de 90 minutos centrada en la violación de expectativas o a una condición de control sin centrarse en las expectativas. Las evaluaciones tuvieron lugar antes de la sesión y una semana después, midiendo los cambios en las respuestas de miedo durante una tarea de visualización basada en un guion y los síntomas de TEPT.

**Resultados:** Utilizando análisis multinivel, no encontramos diferencias entre condiciones. En promedio, las respuestas de miedo a las imágenes y los síntomas de TEPT disminuyeron con el tiempo. La condición de violación de expectativas mostró una mayor disminución en la valoración de la amenaza, lo que pareció mediar la reducción de los síntomas.

**Conclusiones:** No encontramos pruebas de que centrarse explícitamente en la violación de expectativas condujera a efectos superiores inmediatos. Sin embargo, puede conducir a más cambios en las expectativas que podrían afectar la mejora de los síntomas durante un período prolongado. Se necesita más investigación para determinar si es ventajoso enfatizar la violación de expectativas en la terapia de exposición para el TEPT.

## 1. Introduction

Although it has been known for decades that exposure therapy is effective for PTSD (McLean et al., 2022), the discussion about how exposure works is still ongoing. Exposure therapy for PTSD (Foa et al., 2019) consists of systematic and repeated confrontations with (a) the fear-provoking traumatic memories (imaginal exposure) and (b) trauma-related situations, objects, or stimuli that are typically avoided or causing distress (*in vivo* exposure). While many patients with PTSD benefit from exposure therapy, approximately 30% report residual symptoms at a clinical level, indicating that there is room for improvement (Carpenter et al., 2018; Larsen et al., 2019; Springer et al., 2018). To improve efficacy, exposure therapy’s mechanisms of action and how to engage them need to be clarified.

One proposed mechanism is inhibitory learning, which is based on extinction processes (Craske et al., 2008, 2014, 2022). In laboratory settings, during extinction, a fear-eliciting conditioned stimulus (CS) is repeatedly presented in the absence of the unconditioned stimulus (US), leading to a reduction of the conditioned response (CR; i.e. fear). The original fear excitatory association (CS-US) is not forgotten or erased, but rather a second, non-threat association (CS-noUS; i.e. inhibitory association) is learned (Bouton, 1993; Craske et al., 2022), which competes for retrieval with the fear excitatory association. Inhibitory learning refers to the formation of these inhibitory associations. Strengthening these associations and their retrievability during exposure therapy is thought to be a promising approach to increase response rates and to reduce relapse rates.

The inhibitory retrieval model (Craske et al., 2014, 2022) posits that a mismatch between expectancies and outcome drives the formation of inhibitory associations (Craske et al., 2014). This is based on research showing that the strength of new learning was influenced by the magnitude of prediction error (i.e. the discrepancy between expectation and outcome; Rescorla & Wagner, 1972). In the context of exposure therapy for PTSD, expectancies refer to the perceived likelihood that the confrontation with a feared stimulus will lead to a negative outcome (e.g. ‘If I go out on the street, I will be assaulted’). The mismatch between the threat expectancy before exposure and the actual experience during exposure is called expectancy violation. It is proposed that maximally violating expectancies during exposure promotes the learning of inhibitory associations and may thereby optimize treatment efficacy (Craske et al., 2014; Weisman & Rodebaugh, 2018). The inhibitory retrieval model has been mostly tested among individuals with anxiety disorders (Craske et al., 2014; de Jong et al., 2019). In PTSD, it is also theorized to be one of the central principles (Cooper et al., 2017; Craske et al., 2014), but fewer studies have assessed this. How expectancy violation can be promoted during exposure treatment of PTSD is not yet known. The best investigated treatment for PTSD, prolonged exposure (PE; Foa et al., 2019) does not explicitly identify CSs that predict the US nor is the CS-noUS association emphasized after exposure. Possibly, exposure where attention is paid to the identification and non-occurrence of the anticipated negative outcome could enhance exposure outcomes. This could be accomplished by presenting the exposure sessions as ‘experiments’ – to test the hypothesis that the US occurs.

To identify mechanisms of change, two types of studies are needed. Firstly, treatment studies that establish a (temporal) link between the mechanism and treatment outcome, and secondly, experimental studies that manipulate the proposed mechanism (Kazdin, 2007), for instance, by adapting exposure delivery and active elements (Cohen et al., 2023). Thus far, studies that have linked expectancy violation to exposure treatment outcome have yielded mixed results. In PTSD, we found that imaginal exposure led to expectancy violation, yet the degree of expectancy violation was not related to PTSD symptom reduction after treatment (de Kleine et al., 2017). The analysis of a large number of exposure records (*N* = 8,484) from patients suffering from various anxiety disorders, showed that exposure consistently led to expectancy violation and that expectancies changed following exposure (Pittig et al., 2022). Not expectancy violation (i.e. the mismatch between expectancies and outcomes) but rather the expectancy *change* (i.e. the updating of expectancies after their violation) was related to treatment outcome, suggesting that violation is an important step to establish expectancy change, which in turn leads to symptom reduction.

In experimental paradigms wherein expectancy violation was manipulated, findings have also been mixed. An analogue study on interoceptive exposure for panic symptoms showed that an exposure session that continued until expectancies were low (<5%) outperformed a regular exposure session based on fear reduction (Deacon et al., 2013), with the caveat that participants in the experimental group also received more exposure trials, complicating the comparison between conditions. Conversely, two studies on spider phobia and claustrophobia found that the use of cognitive techniques prior to exposure that reduce the perceived likelihood of the aversive outcome (thereby limiting the magnitude of expectancy violation), did not negatively affect exposure outcomes (Buchholz et al., 2022; Krause et al., 2022). Studies testing the effect of exposure therapy delivery with an emphasis on expectancy violation are scarce and most have been carried out in (small) analog samples (Jacoby & Abramowitz, 2016). Given its potential to improve exposure outcomes (Craske et al., 2008, 2014, 2022), it is crucial to investigate whether explicitly focusing on expectancy violation during exposure will enhance outcomes, especially in clinical samples such as PTSD.

The aim of the current study was to examine whether exposure that explicitly focuses on expectancy violation improves the efficacy of PTSD treatment. We carried out a clinical assay (one-session treatment protocol; Rodebaugh et al., 2013). Closely mimicking the timeframe of fear conditioning studies, the clinical assay consisted of one session of exposure therapy followed by a one-week follow up measurement. In a treatment-seeking PTSD sample, we examined whether exposure with an explicit focus on expectancy violation (experimental condition; EXP) led to better outcomes than exposure wherein no explicit attention was paid to expectancies and their violation (control condition; CTL). We assessed pre-to post-intervention changes in fear-related responses (subjective distress and psychophysiology) to a personalized trauma-imagery task, which has shown to be sensitive to change after one exposure session (Tuerk et al., 2018; Wangelin & Tuerk, 2015). We expected that fear-related responses would significantly decrease from pre – to post-exposure session, and that this decrease would be greater in the EXP condition. Furthermore, we examined whether treatment condition affected pre – to post-exposure session change in self-reported PTSD symptoms, and hypothesized that participants in the EXP condition would show greater change. Measuring individualized threat expectancy violations in both conditions was problematic, as it would require identifying expectancies and thereby undermine our manipulation. Therefore, we used a general cognitive measure to assess threat appraisals related to PTSD outcomes. We assessed whether change in threat appraisal mediated intervention effects, and expected that especially in the EXP condition, intervention effects would be driven by change in threat appraisal. Between-condition baseline differences in treatment credibility and expectancy were checked, and to gather information about acceptability of exposure procedures, treatment burden and experience were assessed post intervention.

## 2. Methods

### 2.1. Design

The current study was a clinical assay (one-session treatment protocol) comparing exposure with an explicit focus on expectancy violation (EXP) to a control condition (CTL). A clinical assay has been developed as an alternative to large clinical trials and uses a ‘quick win, fast fail’ approach (Rodebaugh et al., 2013). This paradigm has previously been used in studies aimed at optimizing treatment for social anxiety disorder, panic disorder, and spider phobia (Davis et al., 2017; Hutschemaekers et al., 2020; Rodebaugh et al., 2013; Salkovskis et al., 1999; Shiban et al., 2013).

Assessments occurred at four timepoints: online questionnaires a week before the first lab visit (T0), the lab visit (T1), an exposure session the same day (T2), and a final lab visit a week later (T3). This study was approved by the Medical Ethical Committee of Leiden University Medical Centre (NL73480.058.20).

### 2.2. Randomization

Participants were randomly allocated to a treatment condition (i.e. EXP vs CTL). Randomization was carried out through a computer-generated randomization list by an independent researcher. Randomization was stratified on PTSD symptom severity, i.e. low vs. high scores on the PCL-5 (cut-off = 50). Participants were not blind to treatment conditions. However, all treatment information was presented in such a way that the direction of the hypotheses was unclear.

### 2.3. Participants

Participants were recruited from two outpatient clinics specializing in the treatment of trauma-related disorders from November 2020 to December 2022. Inclusion criteria were: (1) A current PTSD diagnosis (DSM-5 criteria); (2) self-reported PTSD symptoms above clinical cut-off (i.e. PCL-5 score > 31); (3) at least one specific memory related to the index trauma; (4) age between 18 and 70 years. Exclusion criteria were: (1) current trauma-focused treatment; (2) significant suicidal ideations/serious self-injurious behavior or enactment of suicidal behaviors or serious self-injurious behavior within 3 months before intake; (3) intellectual disability; (4) severe substance use disorder; (5) somatic illness that interfered with exposure interventions or planned assessments; (6) pregnancy; (7) unstable regimen of psychotropic medication within 6 weeks before enrollment; (8) no commitment to refrain from using sedative medication/alcohol on the assessment days; and (9) insufficient command of Dutch language. Informed consent was obtained from all patients. An a-priori power analysis revealed that 52 participants would suffice to detect large effects with a power of .80 and alpha of .05. We decided to include 60 participants.

### 2.4. Exposure session

All participants received one 90-minute session of standardized exposure therapy conducted by a therapist trained in exposure therapy for PTSD (MA level or higher). The rationale was delivered through a 3-minute animation video that the therapist and the patient watched together. The traumatic event targeted (i.e. target trauma) in the exposure sessions was similar to the event described in the trauma-imagery (see below).

The exposure session in the EXP condition employed an inhibitory learning-based approach, where expectancies and their non-occurrence were explicitly formulated and tracked, using the session form introduced by Craske et al. (2014). The session started with psycho-education, which focused on the process of expectancy violation. Feared negative outcomes (i.e. the US) were identified using the session form (‘What are you most worried will happen?’) and were modified as needed to ensure they were specific and testable during the exposure session (e.g. ‘I will suffocate’). Participants also provided a likelihood rating for this outcome. Outcomes related to intolerable distress (‘I will be unable to function’) were further specified to testable outcomes through tests of goal-directed actions (e.g. completing a simple task; see also Craske et al., 2022). The patient then continued with the exposure exercise (‘What is your goal?’). The exposure exercises were designed in a way that allowed the feared outcome to be tested. Examples of feared outcomes were ‘not being able to stop hyperventilating’, ‘losing control’, ‘fainting’, and ‘dying’. The first exercise was the same for every participant (recounting the event from beginning to end twice, with a suggested duration of approximately 20 minutes). Therapists were allowed to deviate from the number of repetitions if patients required more or less time to complete their recounts. In follow-up exposure exercises, the exposure target was the stimulus thought to be most associated with the feared outcome in session (i.e. principal CS), for instance, recounting a specific part of the traumatic memory. Attention was paid to the removal of safety signals, as these eliminate or decrease expectancies and thereby minimize expectancy violation. For instance, the therapist would leave the room while the patient completed the exposure exercise if this would increase the perceived likelihood of the occurrence of the feared outcome. Crucially, after each exposure exercise, attention was paid to the recognition and the non-occurrence of the anticipated negative outcome, to promote consolidation of the new learning (i.e. the CS-noUS association). Following the Craske et al. (2014) session form, participants answered the following questions: ‘Did what you were most worried about occur’? (dichotomous, yes/no), ‘How do you know?’, and ‘What did you learn?’. Therapists were instructed to complete a minimum of three exposure exercises in 60 minutes, with a minimum of 45 minutes of exposure. Prior to the start of exposure, expectancies were generally high (*M* = 67.9, *SD* = 22.7). Expectancies were violated in 81 of the 88 exposure exercises (92.0%).

The exposure session in the CTL condition employed a habituation-based approach (Foa et al., 2019). The session started with psycho-education, which was focused on habituation and emotional processing. No anticipated negative outcomes were identified. In-session exposure was prolonged, i.e. participants were asked to recount the traumatic event repeatedly and as vividly as possible for 60 minutes. Following initial repetitions, therapists guided patients towards hotspots. After the prolonged exposure, the experience was processed, during which attention was paid to the in-session distress levels, experiences, and thoughts or potential novel insights about the trauma. As opposed to the EXP condition, no expectancy-based session form was used in this condition, as tracking of expectancies in-session would automatically emphasize expectancies and their non-occurrence. Therapists were instructed to complete a minimum of 45 minutes of prolonged exposure.

Treatment adherence was high. All sessions included standardized psycho-education (specific for both conditions) and imaginal exposure. In all EXP-sessions, hypothesis-testing mini experiments were conducted, where 28 sessions (90.3%) contained three or more separate exercises, two sessions (6.5%) contained two exercises, and one contained only one exercise (3.2%). Twenty-seven of the CTL sessions (93.1%) contained a minimum of 45 minutes of imaginal exposure. One of the participants who received less than 45 minutes imaginal exposure dropped out of the treatment and the study. The other participant received 37 minutes of imaginal exposure due to low SUD levels (three consecutive SUDs of 0). To assess potential contamination between conditions, we randomly selected 20% of CTL treatment sessions to verify whether therapists refrained from addressing expectancies. All therapists consistently adhered to the CTL protocol by not addressing expectancies or their violation.

### 2.5. Trauma imagery

We used a similar imagery task as described in previous studies (Tuerk et al., 2018; Wangelin & Tuerk, 2015). The trauma-imagery task has been shown to be a realiable and standardized way to measure physiological reactivity to trauma-reminders (Pineles et al., 2013; Pole, 2007). During T1, the researcher interviewed the participant and gathered information about the Criterion-A traumatic target event (Foa et al., 2007; Wangelin & Tuerk, 2015). Participants were asked to provide sensory information, as well as their thoughts and feelings experienced during the target traumatic event. This information was then incorporated in a 3-minute personalized trauma script, which the researcher constructed and recorded in another room. For baseline measurement, a standardized 9-minute neutral script was used, in which the arrival to a fictional museum was described. All scripts were recorded by the same female researcher. Both the neutral and the personalized script started with the following sentence: ‘Imagine, as vividly as possible, the following scene … ’.

The imagery task itself took place in a therapy room. The scripts were played on a computer and presented through noise-cancelling headphones. The participant was instructed to sit still in a comfortable position with both feet on the ground while listening to the scripts. Participants were asked to close their eyes if possible. Some participants indicated this was too difficult and kept their eyes open. The participant first listened to the neutral script, followed by the personalized trauma script. The imagery task was administered at T1 and at T3.

### 2.6. Measures

#### 2.6.1. Imagery task

**Psychophysiology.** Heart rate (HR) and skin conductance (SC) were continuously measured during the imagery task using the VU Ambulatory Monitoring System (VU-AMS; de Geus et al., 1995) and data were stored using the Data Analysis and Management Software (VU-DAMS). To measure HR, three single-use Ag/AgCl (pre-gelled with isotonic gel) electrodes were placed on the chest (beneath right collarbone, on the left bottom rib, beneath the right bottom rib). To measure SC, two single-use Ag/AgCl electrodes were placed on the inside of the intermediate phalanges of the middle and index fingers of the non-dominant hand. We used the PhysioDataToolbox (Version 0.6.3) to pre-process and clean the HR and SC data (Sjak-Shie, 2022). We applied an ECG signal analyzer to the raw ECG data with a 1 Hz high-pass filter and a 50 Hz low-pass filter. The R-peaks were detected automatically (with a minimum R-peak value of 0.5 mV and a minimum distance between R-peaks of 0.3s). We applied an SC signal analyzer to the raw SC data with a low-pass filter with a cut-off of 2 Hz. HR and SC data were inspected visually and corrected manually in case of artifacts and/or misidentified R-peaks. Following previous studies (Castro-Chapman et al., 2018; Goodman & Griffin, 2018; Kearns & Engelhard, 2015; Tuerk et al., 2018; Wangelin & Tuerk, 2015), mean HR (beats-per-minutes or BPM) and mean skin conductance level (SCL; micro Siemens) were calculated for both scripts (neutral and trauma) and both timepoints (T1 and T3). Following Wangelin and Tuerk (2015), physiological reactivity (HR-R and SCL-R) was calculated as the difference in mean HR and mean SCL between the neutral script (first three minutes) and the trauma script (i.e. mean trauma script – mean neutral script).

**Subjective distress.** Subjective distress was measured using the 0–100 Subjective Units of Distress Scale (SUDS; Wolpe, 1990). In line with the Prolonged Exposure protocol (Foa et al., 2019), the patient first identified SUDS anchor points at 0, 25, 50, 75 and 100. SUD start, peak and – end scores were collected during the neutral script and the trauma script. We used the SUD_peak_ scores during the trauma scripts at T1 and T3 to assess pre – to post intervention change in subjective distress.

#### 2.6.2. Self-report clinical measures

**Self-reported posttraumatic stress symptoms.** Symptoms were measured with the weekly version of the PTSD Checklist for DSM-5 (PCL-5; Blevins et al., 2015). The PCL-5 is a 20-item self-report questionnaire. Items are scored on a 5-point Likert scale, ranging from 0 (not at all) to 4 (extremely). PCL-5 total scores range from 0 to 80. The PCL-5 is considered to have good psychometric properties. The PCL-5 was administered at T0 and T3. Internal consistency at T0 was good (α = .86).

**Posttraumatic cognitions.** Posttraumatic cognitions were measured with the Posttraumatic Cognition Inventory (PTCI; Foa et al., 1999). The PTCI is a 36-item questionnaire, where items are scored on a 7-point Likert scale. The total PTCI score ranges from 33 to 231. The psychometric properties are considered to be good and internal consistency at T0 was excellent (α = .95).

**Life events.** The Life Events Checklist for DSM-5 (LEC-5; Weathers et al., 2013) was used to assess traumatic life events that were experienced in our sample and was administered at T0. The questionnaire consists of 16 items on potential traumatic events.

**Threat appraisals relevant to PTSD** were measured via 24-items measure of concern about negative, concrete, outcomes that might happen when confronted with a trauma-reminder (e.g. ‘not being able to stop crying’, ‘getting a heart attack’, ‘becoming a victim again’). The wording of the items was based on the Appraisal of Social Concerns questionnaire (ASC; Telch et al., 2004), a reliable and valid questionnaire that assesses specific threat appraisals in social anxiety disorder, and adapted to PTSD-related threat appraisals (based on patient data from a previous study, Oprel et al., 2021, and expert consultation). For each item, participants were instructed to rate how concerning an outcome would be when confronted with a trauma-reminder, ranging from 0 (not at all) to 100 (extremely). The total score was calculated by averaging the item ratings, where a higher score reflected a higher concern for threatening outcomes. Threat appraisals were measured at T1 and at T2 (at the end of the exposure session). At T1, participants had a mean score of 37.5 (*SD *= 18.4, range: 2.1-79.6). Most participants (91.7%) had at least one item where they had an concern equal to or above 60. The internal consistency was excellent (α = .91). Threat appraisals at T1 had a medium, significant correlation with the baseline PCL-5 (*r* = .37, *p *= .004) and the PTCI (*r *= .41, *p *< .001). All items, including their descriptives, can be found in the supplement (S1).

#### 2.6.3. Treatment measures

**Treatment credibility and expectancy.** The credibility and expectancy of the exposure session was measured with the 6-item Credibility/Expectancy Questionnaire (CEQ; Devilly & Borkovec, 2000), adjusted for PTSD. The first three items assess treatment credibility and the others assess expectancy. Four items are rated on a 9-point scale and two are rated from 0-100%. In order to make one composite score for each scale (credibility and expectancy), items were first normalized (i.e. each item ranging from 0 to 1), before summing them to create total scores. Total scores for both scales range from 0 to 3. Higher scores reflect higher credibility and expectancy. The psychometric properties of the CEQ are good (Devilly & Borkovec, 2000). The CEQ was administered after psycho-education and before the start of exposure (i.e. during T2).

**Patient – and therapist experience and treatment burden.** Using similar procedures as a previous study (van den Berg et al., 2016), patient and therapist experience was assessed with the following question: ‘How do you look back on the exposure session?’, using a VAS with a range from 0 (‘negative’) to 100 (‘positive’). Treatment burden was assessed with the following question: ‘How burdensome did you find the exposure session?’, using a VAS with a range from 0 (‘not at all’) to 100 (‘extremely’). For therapists, these questions were asked directly after the exposure session (T2). For patients, these questions were asked at the one-week follow-up (T3).

### 2.7. Procedure

Eligible participants were patients with PTSD who were about to start trauma-focused treatment. In – and exclusion criteria were checked by a researcher. DSM-5 PTSD diagnosis was ascertained by clinical interview by the intaker of the treatment facility, either by CAPS-5 (Boeschoten et al., 2018) or SCID-5-S (Arntz et al., 2017). Participants were randomized to one of the two treatment conditions.

Participants filled out the online baseline questionnaires (T0) at some point during the week preceding the on site baseline assessment. The baseline assessment (T1) started with obtaining written informed consent. Participants then filled out the threat-expectancies questionnaire and they subsequently did the imagery task. After approximately a 30-minute break, the participants received one standardized exposure session (T2). All exposure sessions were audio-recorded to control for treatment integrity. After the rationale of the exposure session was explained, participants were asked to fill out the credibility/expectancy questionnaire (CEQ; Devilly & Borkovec, 2000) to assess treatment expectancy and rationale credibility. At the end of the session, participants rated their threat-expectancies again. Approximately one week after the exposure session (*M* = 7.9, *SD* = 2.7), the follow-up assessment took place (T3). During this assessment, participants completed another set of questionnaires and the imagery task. Approximately one day to one month after participating in this study (T3), participants continued with trauma focused treatment at the treatment site.

### 2.8. Statistical analyses

As the treatment data (CEQ, experience and burden) were non-normally distributed, we conducted a non-parametric Mann Whitney U test to test between-condition differences in these measures. Following Wangelin and Tuerk (2015), before carrying out our main analyses, we checked whether participants showed significant increases (i.e. reactivity) in fear levels and physiology (HR and SC) during the imagery task (i.e. manipulation check), by conducting three paired sample t-tests, with HR, SC and SUD as the dependent variable and script type (neutral script and trauma script) as the independent variable.

To assess the effect of condition on physiological reactivity and distress during the imagery task across time (primary aim), we conducted a multilevel mixed model analysis. Outcome measures, HR-R, SCL-R, and SUD_peak_, were entered as the dependent variable in three separate models. Time (T1 and T3) and its interaction with treatment condition (EXP vs. CTL), were entered as independent variables. We used a similar multilevel model to assess the effect of condition and time on self-reported PTSD symptoms (PCL-5). Following recommendations from Fitzmaurice et al. (2012), as this study is a randomized trial and we can therefore assume that conditions are similar at baseline, we excluded the main effect of condition from the multilevel models to increase power. To control for multiple testing, we applied the Benjamini-Hochberg procedure (or false discovery rate; FDR) on these multilevel analyses. We assessed whether threat appraisal change differed between the two conditions, through a multilevel model with condition and time as the independent variables and threat appraisal as the dependent variable. Finally, we assessed whether threat appraisal measured at T2 mediated the relationship between condition and exposure outcomes measured at T3, by running separate mediation models for each outcome measure. Following recommendations by Hayes and Rockwood (2017), all mediation models controlled for baseline (T1) levels of threat appraisal and outcome measures.

Multilevel analyses were tested with maximum likelihood estimation using the lme4 package (v1.1-28; Bates et al., 2015) in R (Version 4.0.1). Mediation analysis were carried out using Hayes’ PROCESS macro in SPSS (Hayes, 2017). A bias-corrected bootstrapping was used (10,000 iterations) to obtain 95% confidence intervals (CIs) to infer statistical significance (the CIs do not include zero). Other analyses were conducted using SPSS (v.27). Alpha levels were set at 0.05 (two-sided). The data-analysis plan of this study was registered at OSF (Center for Open Science; Kooistra et al., 2024).

## 3. Results

### 3.1. Participants

**Figure 1.**
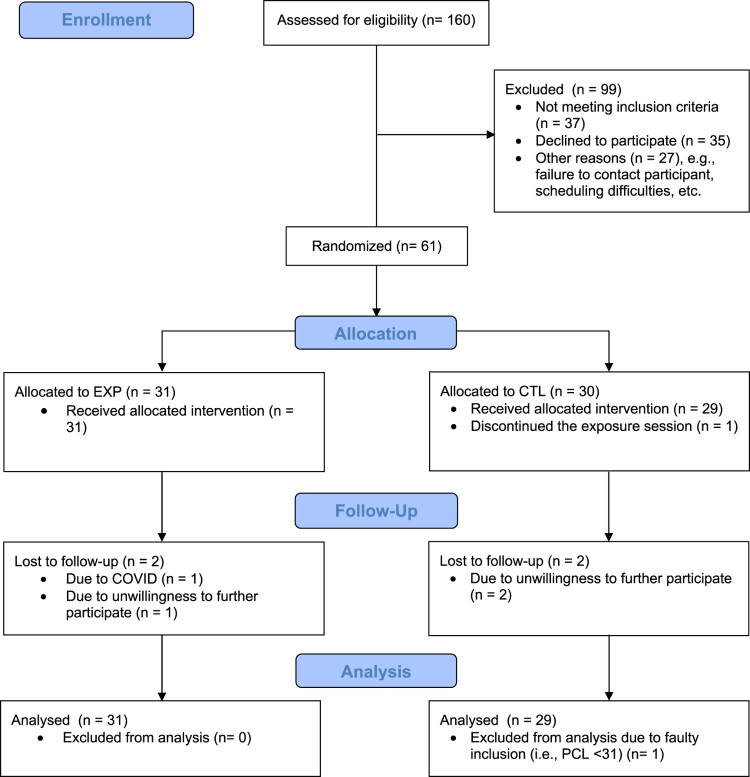
CONSORT participant flow diagram.

The sample consisted of 60 participants (*M*_age_ = 39.7; *SD*_age_ = 12.5), including 41 women, 18 men and one non-binary person. See Figure 1 for the study flowchart. One participant was replaced after receiving the intervention, as this participant failed to fill out the online questionnaires prior to the lab visit, and during the lab visit we found out that this participant did not meet inclusion criteria (i.e. the PCL-5 total score was below 31). One participant in the CTL condition discontinued the exposure session, but all other participants received and finished the exposure session (i.e. the intervention). In both conditions, two participants were lost to follow-up. One of these participants was unable to attend the second study visit (T3) as this person was infected by the corona virus and lock-down rules at the time prohibited in-person meetings. This participant did fill out the follow-up questionnaires (T3) at home. The other three participants did not want to continue with the study.

The sample characteristics are listed in Table 1. As assessed with the LEC-5, fifty-three participants (88.3%) reported a direct experience or witnessing sexual assault and 54 participants (90.0%) reported physical violence. Participants reported to have directly experienced or witnessed an average of 8.4 potentially traumatic events (*SD *= 4.6). No significant between-condition differences were found at baseline.

**Table 1.** Baseline characteristics of participants.

	Total (*N* = 60)	EXP (*n* = 31)	CTL (*n* = 29)
	*n* (%)	*n* (%)	*n* (%)
Gender (woman)	41 (68.3)	22 (71.0)	19 (65.5)
Education (high)^a^	19 (31.6)	7 (22.6)	12 (41.3)
Cultural background (non-Western)^b^	16 (26.7)	8 (25.8)	8 (27.6)
Work/occupation			
Employed	16 (26.7)	7 (22.6)	9 (31.0)
Student	3 (5.0)	2 (6.5)	1 (3.4)
Incapacitated/on disability	18 (30.0)	8 (25.8)	10 (34.5)
Unemployed	20 (33.3)	12 (38.7)	8 (27.6)
Retired	3 (5.0)	2 (6.5)	1 (3.4)
Target trauma			
Sexual abuse as child	24 (40.0)	11 (35.5)	13 (44.8)
Sexual abuse as adult	10 (16.7)	5 (16.1)	5 (17.2)
Physical abuse as child	13 (21.7)	7 (22.6)	6 (20.7)
Physical violence as adult	11 (18.3)	6 (19.4)	5 (17.2)
Deathly accident	2 (3.3)	2 (6.5)	0 (0.0)
	*M (SD)*	*M (SD)*	*M (SD)*
Age	39.7 (12.5)	39.7 (13.7)	39.6 (11.3)
PTSD severity (PCL-5)	55.2 (10.8)	55.5 (12.8)	54.9 (8.4)
Negative cognitions (PTCI)	157.3 (35.0)	162.1 (38.5)	152.1 (30.7)

Note*.* EX*P* = experimental condition; CTL = control condition; PCL-5 = PTSD Checklist for DSM-5; PTCI = Posttraumatic Cognitions Inventory.

^a^ High education = higher vocational education or university.

^b^ Non-Western cultural background = at least one parent who was not born in a Western country.

### 3.2. Treatment credibility and expectations

On average, participants thought that the provided treatment was quite credible (*M = *2.2, *SD *= 0.5), with no between condition differences (*U = *405.5, *p *= .518). Their expectations of the treatment were more towards the positive end of the scale (*M = *1.9, *SD *= 0.5), and did not differ significantly between conditions (*U* = 413.5, *p *= .560).

### 3.3. Physiological reactivity during imagery task

At T1, participants showed a significant increase in HR from the neutral script (*M *= 74.3, *SD* = 10.2) to the trauma script (*M *= 77.8, *SD* = 9.8), *t*(56) = −5.29, *p* < .001, Cohen’s *d *= −0.70; HR reactivity (HR-R), *M* = 3.3, *SD* = 5.2. Participants also showed a significant increase in SCL from the neutral script (*M *= 7.8, *SD *= 4.8) to the trauma script (*M *= 8.1, *SD* = 4.7), *t*(56) = -.49, *p* = .003, albeit to a weaker extent (Cohen’s *d *= −0.37); SCL reactivity (SCL-R), *M *= 0.4, *SD *= 1.3. Peak distress levels (SUD_peak_) strongly and significantly increased from the neutral (*M *= 38.6, *SD*  = 27.7) to the trauma script (*M *= 73.3, *SD* = 23.4), *t*(59) = −11.275, *p* < .001, Cohen’s *d *= −1.52.

### 3.4. Pre – to post exposure session change on outcome measures

**Figure 2.**
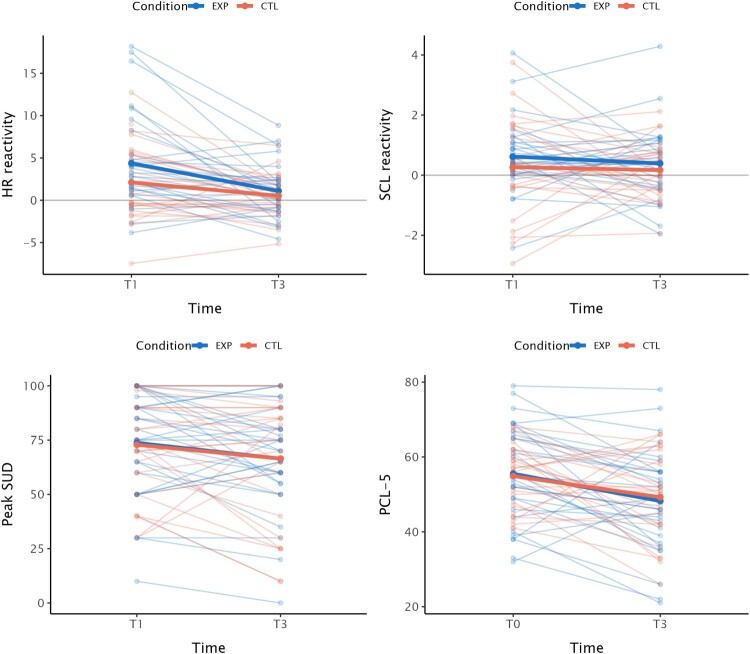
Changes in outcome measures across time per condition.

The change of the outcome measures from pre – to post exposure session can be found in Table 2. Mean scores and participant’s individual trajectories are shown in Figure 2. For the primary outcome measures (i.e. fear responses to the trauma script), no significant interaction effects of time and condition were found, indicating that change from T1 to T3 on fear responses to the trauma script was similar between conditions. We found a significant time effect for HR-R, *b* = −2.43, *SE* = 0.65, *t *= −3.72, *p* < .001, Cohen’s *d* = −0.88, and SUD_peak_, *b* = −6.71, *SE* = 2.98, *t *= −2.25, *p* = .028, Cohen’s *d* = −0.51, indicating that these outcome measures significantly decreased from pre-exposure session to post-exposure session. Effect sizes were medium to large. No significant time effect was found for SCL-R, *b* = −0.10, *SE* = 0.21, *t *= −0.47, *p* = .640. The results of the multilevel model analyses with random intercepts are shown in Table 3.

**Table 2.** Change in outcome measures from pre- to post exposure session.

	Total		EXP		CTL	
	*N*	*M* (*SD*)	*n*	*M* (*SD*)	*n*	*M* (*SD*)
HR-R						
T1	58^a^	3.3 (5.2)	31	4.4 (5.6)	27^a^	2.1 (4.5)
T3	55^b^	0.8 (2.9)	28^b^	1.1 (3.2)	27	0.5 (2.5)
SCL-R						
T1	60	0.4 (1.3)	31	0.6 (1.2)	29	0.3 (1.5)
T3	55^b^	0.3 (1.1)	28^b^	0.4 (1.3)	27	0.2 (0.9)
SUD_peak_						
T1	60	73.3 (23.4)	31	73.7 (24.3)	29	72.9 (22.8)
T3	56	66.5 (26.3)	29	66.4 (24.2)	27	66.5 (28.9)
PCL-5						
T0	60	55.2 (10.8)	31	55.5 (12.8)	29	54.9 (8.4)
T3	57	48.7 (12.5)	30	48.2 (13.7)	27	49.3 (11.2)
Threat appraisal						
T1	59	37.5 (18.4)	30	41.3 (20.5)	29	33.4 (15.2)
T2	59	27.9 (18.6)	30	27.4 (21.0)	29	28.5 (16.1)

Note*.* EX*P* = experimental condition; CTL = control condition; HR-R = Heart rate reactivity during imagery; SCL-R = Skin conductance level reactivity during imagery; SUD = subjective units of distress; PCL-5 = PTSD Checklist for DSM-5; T0 = baseline questionnaire (online); T1 = baseline assessment (on site); T2 = at the end of the exposure session; T3 = one week after the exposure session.

^a^ HR data was missing for two participants at T1 (both in CTL condition) due to equipment problems that occurred during the assessment.

^b^ Physiological data (HR and SCL) was missing for one participant at T3 due to equipment problems.

**Table 3.** Outcomes multilevel models.

	B	SE	t	*p*	p*	d
HR-R						
Intercept	5.89	0.99	5.93	<.001	.002	
Time	−2.43	0.65	−3.72	<.001	.002	−0.88
Time*Condition	−0.26	0.55	−0.48	.635	.731	−0.10
SCL-R						
Intercept	0.61	0.31	1.95	.054	.079	
Time	−0.10	0.21	−0.47	.640	.731	−0.11
Time*Condition	−0.14	0.16	−0.90	.369	.492	−0.20
Peak SUD						
Intercept	79.74	4.79	16.66	<.001	.002	
Time	−6.71	2.98	−2.25	.028	.044	−0.51
Time*Condition	0.55	3.15	0.17	.863	.863	0.03
PCL-5						
Intercept	61.78	2.64	23.43	<.001	.002	
Time	−6.79	1.71	−3.97	<.001	.002	−0.91
Time*Condition	0.43	1.52	0.29	.776	.828	0.06
Threat appraisal						
Intercept	57.61	4.57	12.61	<.001	.002	
Time	−15.09	2.23	−6.78	<.001	.002	−1.78
Condition	−19.30	6.51	−2.97	.004	.007	−2.27
Time*Condition	10.19	3.16	3.23	.002	.004	0.85

Note*.* HR-R = Heart rate reactivity during imagery; SCL-R = Skin conductance level reactivity during imagery; SUD = subjective units of distress; PCL-5 = PTSD Checklist for DSM-5; p* = corrected *p*-value with False Discovery Rate.

**Figure 3.**
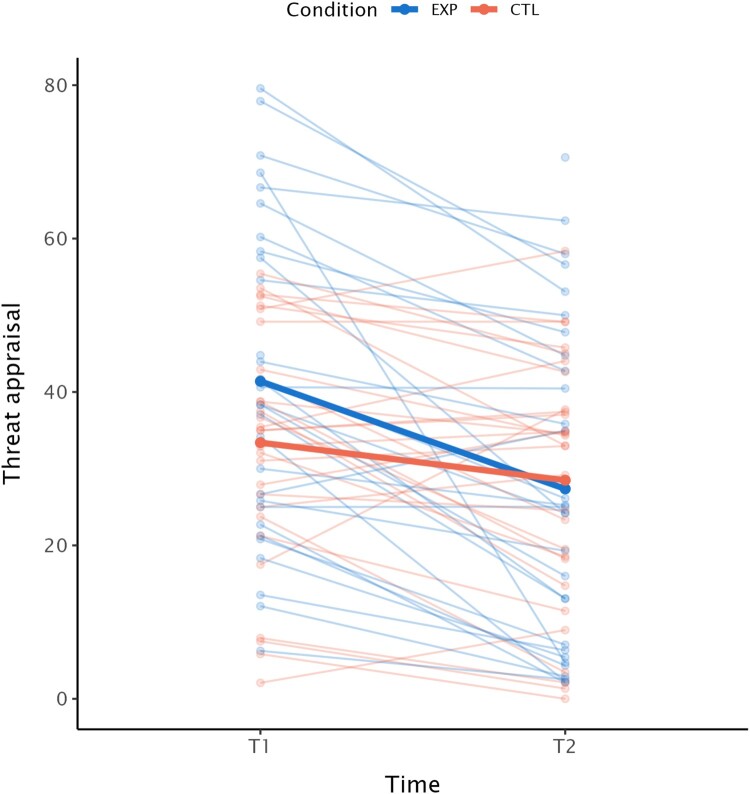
Threat appraisal across time per condition.

Regarding the secondary outcome measure, we found no significant interaction effects of time and condition, indicating that change in PTSD symptomatology from T1 to T3 was similar between conditions. As threat appraisal did seem to differ between conditions at baseline, we added condition as a main effect to this model as well. We found a significant time effect for the PCL-5, *b* = −6.79, *SE* = 1.71, *t *= −3.97, *p* < .001, Cohen’s *d* = −0.91. We found a significant effect of time on threat appraisal, *b* = −15.09, *SE* = 2.23, *t *= −6.78, *p* < .001, and a significant interaction of time by condition, *b* = 10.19, *SE* = 3.16, *t *= 3.23, *p* = .002, Cohen’s *d* = 0.85, suggesting that those who were in the EXP condition had a larger decrease in threat appraisal compared to those in the control condition (see also Table 2 and Figure 3). We have reported the unadjusted *p*-values in this section, the corrected *p*-values can be found in Table 3.

### 3.5. Threat appraisal as mediator of exposure effects

We expected that especially in the EXP condition, intervention effects would be driven by change in threat appraisal. In line with our hypotheses, the indirect effect of condition on both HR-R and PCL-5 through threat appraisal was significant, *b* = 0.56, *SE* = 0.27, 95% CI [0.01, 1.09] and, *b *= 2.60, *SE *= 1.27, 95% CI [0.69, 5.60], respectively. This suggests that the effect of condition on HR-R and PCL-5 was mediated by (lower) threat appraisal following exposure. The indirect effect of condition on SUD_peak_ through threat appraisal (*b *= 3.61, *SE *= 2.19, 95% CI [−0.13, 8.49]) was non-significant. We did not assess a mediation with SCL-R, as there was no time effect with this outcome variable. Complete mediation outcomes (i.e. a, b, c and c’ paths) can be found in the supplement (S2).

### 3.6. Treatment acceptability

On average, based on a 0–100 VAS scale, patients felt relatively positive about the exposure session, with no significant differences between conditions (EXP: *M *= 69.1, *SD *= 21.2; CTL: *M *= 66.3, *SD *= 25.6; *U* = 389.0 *p *= .859). Patients also found the exposure session quite burdensome, again with no significant differences between conditions (EXP: *M *= 83.9, *SD *= 19.1; CTL: *M *= 78.0, *SD *= 24.1; *U* = 443.0, *p *= .398). An open-ended question inquiring about the aspects that participants found most burdensome showed that participants mostly referred to exposure to the trauma memory (and not to specific treatment elements, per se).

As for the therapists, no significant between condition differences were found for how positively (EXP: *M *= 67.6, *SD *= 21.2; CTL: *M *= 73.8, *SD *= 18.0; *U* = 361.0, *p *= .193) nor how burdensome (EXP: *M = *42.7, *SD *= 26.1; CTL: *M = *33.2, *SD *= 22.8; *U* = 555.0, *p *= .120) they evaluated the session.

## 4. Discussion

We found no differences in outcomes between two exposure conditions with or without an explicit focus on expectancy violation in a clinical sample of treatment-seeking PTSD patients using a single-session paradigm. Exposure outcomes were assessed through fear-related responses (heart rate reactivity, skin conductance reactivity and peak subjective distress) to a personalized imagery task. The secondary outcome was self-reported PTSD symptoms. Most of our participants had PTSD related to interpersonal violence. All measures, except skin conductance reactivity, decreased from pre – to post exposure session. We found some evidence that threat appraisals mediated exposure effects, indicating that more so in the expectancy violation condition, reduction in fear and PTSD symptoms was partly driven by a reduction in threat appraisal. Finally, therapists’ and patients’ experiences of the exposure sessions did not differ between conditions.

Based on the suggestion from the inhibitory retrieval approach (Craske et al., 2014, 2022) that a focus on prediction error might enhance exposure’s efficacy, we expected that the expectancy violation condition would lead to more symptom reduction, but, surprisingly, we found no differences between conditions. Our finding also contrasts with an earlier study where exposure outcomes were superior when exposure continued until threat expectancies were low (Deacon et al., 2013). However, in line with our null-finding, other studies showed that the magnitude of expectancy violation was not associated with enhanced extinction learning or exposure outcomes (Buchholz et al., 2022; De Jong et al., 2023; Krause et al., 2022; Stemerding et al., 2023). Furthermore, a wealth of studies demonstrate the efficacy of Prolonged Exposure, a specific exposure therapy protocol for PTSD without an explicit focus on expectancy violation (McLean et al., 2022). In the current study, we do not find evidence that emphasizing the identification and the non-occurrence of negative expectancies during exposure leads to enhanced immediate PTSD-related treatment outcomes. As we used a single-session paradigm, we cannot rule out the possibility that one session was not sufficient to elicit longer term change.

Overlapping mechanistic constructs and varying construct operationalizations across the field make it difficult to study mechanisms (Benito et al., 2024; Cohen et al., 2023). We tried to elucidate how treatment delivery affected the the mechanism of expectancy violation. Although we did not find significant differences between conditions in symptom reduction, our mediation findings suggest that especially in the EXP condition, intervention effects were driven by change in threat appraisal. More specifically, participants in the EXP condition showed a greater decrease in concern about potential PTSD-related negative outcomes, leading to more symptom reduction. These findings could suggest that focusing on threat expectancies and their violation during exposure enhances expectancy change (perhaps driven by enhanced awareness, see also Stemerding et al., 2023), which could subsequently drive symptom improvement (Pittig et al., 2022). Although, given unexpected baseline differences in threat appraisal between conditions, the alternative explanation that this reflects regression to the mean cannot be ruled out. As our control condition was not outperformed, alternative mechanisms might have played a bigger role here, such as distress habituation and more general cognitive change, i.e. cognitive change that is not related to CS-US predictions, such as negative views and judgments about oneself or the meaning of the trauma (Cooper et al., 2017). The effectiveness of treatment deliveries may also vary based on individual factors, such as differences in symptom presentations (e.g. varying levels of persistent negative beliefs or self-blame). Future research with larger sample sizes should assess multiple mediators simultaneously and assess what works best for whom.

It remains unclear whether it is advantageous to emphasize expectancies and their non-occurrence during (imaginal) exposure for PTSD. The inhibitory retrieval approach (Craske et al., 2022) attempts to target the principal CS-US association. As such, accurately identifying the most feared outcome (i.e. the US) is crucial. This requires that patients both recognize this outcome and can clearly articulate it. However, some patients have difficulty identifying their greatest fear, may have long-term feared outcomes that are untestable, or may have feared outcomes related to the inability to tolerate distress (Jacoby & Abramowitz, 2016; Scheveneels et al., 2019). This was also true for some patients in our study and may be more prevalent in complex clinical populations such as obsessive compulsive disorder (OCD) or PTSD, in comparison to specific phobia. Additionally, exposure with an explicit focus on expectancy violation may work better for ‘in vivo’ than imaginal exposures, as expected outcomes are usually more concrete and easier to test in in vivo exposures (e.g. ‘I will be assaulted again when going to crowded places). Undoubtedly, cognitive changes, including the updating of expectancies, are central to the effectiveness of exposure therapy for PTSD (Brown et al., 2019). However, as suggested by EPT (Cooper et al., 2017; Foa & Kozak, 1986), these changes may also occur implicitly, with patients modifying their cognitions at a level that does not require conscious awareness.

The current study has a number of limitations, including the single-session paradigm. A benefit of this paradigm is that we were able to isolate the effect of the manipulation more easily than in a large longitudinal trial with more confounding factors (e.g. doing homework, external stressors during the timeframe of treatment, etc.). However, we were unable to assess the effect over an extended period. Some expectancies may only be violated over repeated exposures. For instance, some patients are afraid that repeated exposure will lead to a mental catastrophe which makes them unable to take care of children or function at work. A full-scale RCT should be carried out, wherein the inhibitory retrieval model’s posited strategies to enhance exposure outcomes are tested. Furthermore, this single session may have been insufficient for some patients to achieve meaningful improvements, which hinders comparisons between the delivery method of this session. It should also be noted that, on average, physiological reactivity at pretreatment was relatively low and some patients in our sample showed a blunted physiological response to the script-driven imagery, which may reflect dissociation (Carpenter et al., 2024; Sack et al., 2012). For these patients, a decrease of reactivity from pre to post treatment would actually not reflect better outcomes. Future studies using physiology during trauma-imagery should account for differential responding to trauma reminders in those suffering from PTSD. Crucially, we could not compare in-session threat expectancy violation between conditions, as it would have drawn attention to expectancies in the control group, undermining our manipulation. A more direct measure of the underlying mechanism would have been ideal, but it is still unclear how much simply measuring threat expectancies influences exposure outcomes. In the expectancy violation condition, we used the session form from Craske et al. (2014) to design exposure exercises. However, the form only includes pre-exposure perceived likelihood ratings, preventing us from tracking the degree of violation and its impact on outcomes. For our mediation analysis we used an unvalidated measure to operationalize threat appraisal, based on a validated measure to assess appraisal in social phobia (Telch et al., 2004). The results of our mediation analysis should thus be interpreted with caution. Finally, our study was powered to detect large effects, as these were deemed clinically meaningful, but the study may have been underpowered to detect smaller differences between conditions.

The current study also has several strengths. Our study is the first that directly tests the effect of therapeutic procedures targeting expectancy violation on symptom reduction, while limiting dosage differences between conditions. We assess these effects in a clinical sample representative of routine clinical care (i.e. treatment-seeking PTSD patients). Additionally, few participants were lost to one-week follow-up. To assess exposure outcomes, we used a combination of measures, including self-report and psychophysiology. Finally, we test the acceptability of the exposure conditions, which has not been done previously. A recent meta-analysis suggested that the acceptability of exposure therapy is somewhat lower compared to other psychological interventions for PTSD (Hoppen et al., 2023). Given that exposure is, among others, the most effective treatment for PTSD, gaining insight into the theurapeutic procedures that affect acceptability may be a crucial step in improving its efficacy.

To conclude, we found that exposure with an explicit focus on expectancy violation was not related to better outcomes. We also found that threat appraisal changes upon exposure, and more so in exposure that focuses expectancy violation. This, however, did not immediately transfer to PTSD symptomatology. Future work should address how to operationalize and measure threat expectancies and assess its long-term effects on exposure outcomes. More empirical work is necessary to assess whether the application of the inhibitory retrieval-based approach to exposure for PTSD is beneficial in routine clinical care.

## Supplementary Material

20241218_Expectancy_violation_and_PTSD_Supplement.pdf

## Data Availability

The anonymized data that support the findings of this study are available from the corresponding author, MJK, upon reasonable request.
