# Supplementary material for: Maximizing expectancy violation and exposure outcomes in patients with PTSD
Source: Eur J Psychotraumatol. 2025 Jan 7;16(1):2447183. doi: 10.1080/20008066.2024.2447183 (PMC11721951; doi:10.1080/20008066.2024.2447183)
Supplement: 20241218_Expectancy_violation_and_PTSD_Supplement.pdf [file ZEPT_A_2447183_SM9385.pdf]

Supplement

Maximizing expectancy violation and exposure outcomes in patients with PTSD

Marike J. Kooistra, Maartje Schoorl, Danielle A. C. Oprel, Willem van der Does, and

Rianne A. de Kleine

Contents

Supplement 1. Threat appraisal measure 1
Supplement 2. Mediation analyses.. 2
References 3

Supplement 1. Threat appraisal measure

The instructions and scoring of the threat appraisal measure was based on the Appraisal of Social Concerns questionnaire (ASC; Telch et al., 2004). Similar to the ASC, we chose to ask participants to rate their degree of concern about a negative anticipated outcome, aiming to capture its perceived likelihood and threat, whilst keeping the measure concise and easy to administer. Participants are asked to rate their level of concern for a negative outcome when confronted with a trauma reminder, ranging from 0 ('not at all concerned') to 100 ('extremely concerned'), where a score of 50 represents moderate concern.

Table S1

Overview of TAPS items, including their mean and rank at T1

		M (*SD*)	Rank
1	Screaming	28.1 (31.6)	19
2	Throwing things	30.7 (31.7)	16
3	Vomiting	30.7 (32.3)	16
4	Having a heart attack	19.5 (27.0)	23
5	Becoming a victim again/being in danger	55.5 (37.8)	3
6	Choking	28.7 (32.1)	18
7	Unable to move	41.9 (36.1)	8
8	Fainting	34.8 (30.7)	13
9	Not knowing where I am	39.3 (32.8)	10
10	Hitting or kicking	26.8 (33.1)	20
11	Unable to talk	45.3 (34.1)	7
12	Unable to think (having a blackout)	62.3 (32.4)	1
13	Swearing or cursing	35.0 (36.7)	11
14	Unable to feel anything	53.1 (34.5)	4
15	Hurting myself	41.9 (37.2)	8
16	Wetting or soiling my pants	12.1 (24.2)	24
17	Collapsing	32.6 (31.1)	14
18	Dying	22.3 (32.9)	21
19	Unable to stop crying	48.2 (34.9)	6
20	Speaking gibberish	32.3 (35.1)	15
21	Walking away or running away	50.3 (34.5)	5
22	Hurting someone else	20.4 (30.2)	22
23	Moving uncontrollably	35.0 (35.7)	11
24	Unable to function	57.3 (34.2)	2

Notes. M = mean; *SD* = standard deviation; Rank = relative standing of the item based on highest mean.

Supplement 2. Mediation analyses

Table S2.

Results from mediation of threat appraisal between condition and exposure outcome variables.

	B	SE	*t*	*p*	LLCI	ULCI
Model 1						
C → TA at T2 (a)	9.48	3.27	2.90	**.006**	**2.92**	**16.06**
TA at T2 → HRR at T3 (b)	0.06	.03	1.89	.064	-0.00	0.12
C→ HRR at T3 (c)	0.25	0.73	0.34	.732	-1.21	1.71
C→ HRR at T3 (c’)	-0.31	0.77	-0.40	.692	-1.85	1.23
C→ TA at T2→ HRR at T3 (a*b)	0.56	0.27			**0.01**	**1.09**
Model 2						
C → TA at T2 (a)	8.72	3.13	2.78	**.008**	**2.44**	**15.01**
TE at T2 → SUD at T3 (b)	0.41	0.23	1.80	.077	-0.05	0.87
C→ SUD at T3 (c)	0.80	5.30	0.15	.880	-9.83	11.43
C→ SUD at T3 (c’)	-2.81	5.56	-0.51	.616	-13.97	8.35
C→ TA at T2→ SUD at T3 (a*b)	3.61	2.19			-.13	8.49
Model 3						
C → TA at T2 (a)	9.05	3.18	2.84	**.006**	**2.66**	**15.43**
TA at T2 → PCL5 at T3 (b)	0.29	0.12	2.37	**.022**	**0.04**	**0.53**
C→ PCL5 at T3 (c)	3.32	2.91	1.14	.259	-2.51	9.15
C→ PCL5 at T3 (c’)	0.71	2.99	0.24	.812	-5.30	6.72
C→ TA at T2→ PCL5 at T3 (a*b)	2.60	1.27			**0.69**	**5.60**

Notes. Model 1 is mediation model with heart rate reactivity as the outcome variable; Model 2 is mediation model with SUD peak as outcome variable; Model 3 is mediation model with the PLC-5 as outcome variable; a = effect of X on M; b = effect of M on Y; c = total effect of X on Y; c’ = direct effect of X on Y controlling for M. The standard error and 95% CI for a*b are obtained by bootstrap with 50,000 re-samples. C = Condition; TA = Threat appraisal; HRR = Heart rate reactivity; SUD = subjective units of distress; PCL5 = PTSD symptomatology; T1 = baseline assessment (on site); T2 = at the end of the exposure session; T3 = one week after the exposure session; LLCI = lower bound of a 95% confidence; ULCI = upper bound of a 95% confidence interval; → = affects.

References

Telch, M. J., Lucas, R. A., Smits, J. A. J., Powers, M. B., Heimberg, R., & Hart, T. (2004). Appraisal of Social Concerns: A cognitive assessment instrument for social phobia. *Depression and Anxiety*, *19*(4), 217–224. https://doi.org/10.1002/da.20004
